# Hospital outbreak sustained by *Klebsiella pneumoniae* sequence type 147 co-producing NDM-1 and OXA-48, Rome, Italy, February to March 2025: molecular tracing and control measures

**DOI:** 10.2807/1560-7917.ES.2026.31.10.2500457

**Published:** 2026-03-12

**Authors:** Valerio Capitani, Mariateresa Ceparano, Annalisa Rosso, Guido Antonelli, Maria Augurusa, Valentina Baccolini, Giancarlo Ceccarelli, Maria De Giusti, Claudio Maria Mastroianni, Francesco Pugliese, Federica Sacco, Agnese Viscido, Paolo Villari, Alessandra Carattoli, Carolina Marzuillo, on behalf of the Outbreak Investigation Collaborating Group

**Affiliations:** 1 Department of Public Health and Infectious Diseases, Sapienza University of Rome, Rome, Italy; 2 Department of Molecular Medicine, Sapienza University of Rome, Rome, Italy; 3 Microbiology and Virology Unit, Sapienza University Hospital “Policlinico Umberto I”, Rome, Italy; 4 Sapienza University Hospital “Policlinico Umberto I”, Rome, Italy; 5 Members of the Outbreak Investigation Collaborating Group are listed under Acknowledgements

**Keywords:** Infection prevention control, outbreak investigation, active surveillance, pNDM-MAR, repB(R1701), CTX-M-15 four copies, molecular typing

## Abstract

We describe an outbreak of carbapenemase-producing *Klebsiella pneumoniae* that occurred in February–March 2025 in a tertiary care hospital in Rome, Italy. Ten patients in two adjacent critical care units were found colonised or infected with strains co-producing NDM-1 and OXA-48 carbapenemases. Nine of these had acquired infections in our hospital and were detected through routine or enhanced rectal swab screening. Rapid implementation of infection prevention and control (IPC) measures, including patient cohorting, environmental cleaning and contact surveillance, helped contain transmission. Despite these measures, the outbreak spread across wards, suggesting challenges in timely isolation, patient movement within hospitals and contact precautions in intensive care units. Environmental sampling revealed a single surface contaminated with the outbreak strain. Rapid molecular typing using an in-house nanopore sequencing protocol (NanoTyping), supported by whole genome sequencing, confirmed clonal spread of the high-risk ST147 clone. Phylogenetic comparison with Italian isolates from previous outbreaks indicated regional dissemination of this clone. This outbreak highlights the need to combine genomic surveillance and rapid diagnostics to support real-time response. In settings with high multidrug-resistant organism (MDRO) prevalence, enhanced screening, routine surveillance and rigorous IPC protocols remain critical to prevent and control the spread of emerging high-risk clones in healthcare facilities.

**Key public health message**

**What did you want to address in this study and why?**

Controlling multidrug-resistant organisms (MDRO) in critical care settings poses a notable challenge. Here we describe an outbreak affecting 10 patients in intensive care units of a hospital in Rome, Italy, caused by a *Klebsiella pneumoniae* ST147 clone, identified in February–March 2025, to understand the transmission dynamics of this strain, which had not been previously detected in our setting.

**What have we learnt from this study?**

This study highlights the rapid spread of an NDM-1/OXA-48-producing *K. pneumoniae* clone across intensive care wards, even with contact precaution measures enacted. Active patient screening and rapid molecular typing enabled early outbreak detection and strain discrimination.

**What are the implications of your findings for public health?**

A *K. pneumoniae* ST147 clone, characterised by extensive antimicrobial resistance, is spreading across southern regions in Italy, causing hospital outbreaks. Enhanced screening, environmental monitoring, and strict IPC protocols are essential to contain high-risk MDRO clones. Molecular tools can strengthen local capacities to manage emerging antimicrobial resistance threats.

## Background

*Klebsiella pneumoniae* is a Gram-negative opportunistic pathogen frequently involved in healthcare-associated infections (HAIs), such as pneumonia, bloodstream infections and urinary tract infections [1]. In Europe, Italy reports one of the highest prevalence rates of carbapenem-resistant *K. pneumoniae*, with both endemic circulation and localised outbreaks observed across regions [2]. Although in Italy the most frequent carbapenem resistance sequence type (ST) is ST512-producing *K. pneumoniae* carbapenemase (KPC) [2], relevant outbreaks caused by ST147 New Delhi metallo-beta-lactamase (NDM) producing strains were reported [3]. In recent years, strains expressing NDM or OXA-48-like carbapenemases have emerged as major nosocomial threats because of their high transmissibility, their broad antibiotic resistance and the resulting limited therapeutic options [4]. These multidrug-resistant organisms (MDRO) pose a substantial threat in intensive care units (ICUs), where critically ill patients and the frequent use of invasive devices facilitate rapid transmission [5]. Several different STs producing both NDM and OXA-48-like enzymes are being increasingly reported across Europe [6,7].

## Outbreak detection

Between mid-February and early March 2025, five patients in the sub-intensive care unit (S-ICU) of a tertiary care hospital in Rome, Italy, tested positive for NDM- and OXA-48 co-producing *K. pneumoniae* following routinary screening rectal swabs. The director of the S-ICU reported the detection of the five positive patients with an Official Note to the Hospital Health Directorate, and investigation was immediately initiated, as no *K. pneumoniae* NDM–OXA-48 had ever been reported in that unit before. Within 2.5 weeks, five more positive patients were identified through active surveillance. This study describes the outbreak investigation, active surveillance of contacts, microbiological findings and infection control measures.

## Methods

### Setting and routine surveillance

This outbreak occurred between February and March 2025 in a 16-bed S-ICU of a 1,200-bed tertiary care hospital and involved subsequent transmission to the adjacent 18-bed ICU.

Since April 2016, an active surveillance system for HAIs has been routinely conducted in the ICU by the Department of Public Health and Infectious Diseases of Sapienza University of Rome, Italy. This system, based on protocols from the National Healthcare Safety Network [8] and the European Centre for Disease Prevention and Control (ECDC) [9], involves the diagnosis of HAIs by infectious disease specialists using a combination of clinical, imaging and laboratory criteria. The detailed methodology of the surveillance system has been described previously [10].

Briefly, all patients admitted to the ICU for at least 2 consecutive days are monitored until discharge for catheter-related bloodstream infections (BSIs), ventilator-associated pneumonia, catheter-associated urinary tract infections, BSIs of unknown origin, healthcare-associated pneumonia, and surgical site infections. In addition, routine multidrug-resistant organisms (MDRO) surveillance in both units included weekly rectal swabs. Each unit had dedicated medical and nursing staff, and no equipment was shared.

### Epidemiological investigation and case definition

Cases were defined as patients admitted to the ICU or S-ICU colonised or infected with NDM-producing *K. pneumoniae* between February and March 2025. A strain was considered acquired if the patient tested positive 48 h after admission, even in the absence of screening at the time of admission, while it was considered imported if the patient tested positive within 48 h after admission.

Active surveillance of contacts was initiated when enhanced infection prevention and control (IPC) measures were implemented. All patients who had been cared for by the same healthcare team were classified as contacts, including all individuals admitted to the S-ICU between 15 February (the admission date of the first positive patient) and 7 March (the starting date of the active surveillance). Those discharged from the hospital before its initiation were excluded.

A prospective analysis was conducted to identify incident cases, based on the active surveillance of contacts initiated by the IPC committee. Patients who remained in the S-ICU underwent rectal swab screening every 48 h. Those who had been transferred to other wards were placed in precautionary isolation and screened upon arrival. If the initial test was negative, screening was repeated 7 days after transfer. At the same time, records from the active surveillance protocol regularly implemented in the ICU were assessed both retrospectively and prospectively, to identify additional cases of NDM-producing *K. pneumoniae* diagnoses in that ward. Patients admitted to the ICU who had not been in contact with the cases remained under the routine surveillance programme, which included weekly rectal swab screening.

### Microbiological investigation

#### Sample collection and sources

Clinical specimens were collected either from contacts as part of active surveillance (as previously described), or from patients admitted to the S-ICU and ICU with suspected infections or during routine screening procedures for MDRO control. The strains were collected, isolated as pure cultures, and stored at −80 °C with glycerol for genotypic typing.

Environmental sampling was carried out to identify potential reservoirs of NDM-producing *K. pneumoniae* during the outbreak investigation in the S-ICU, at the request of infection control staff. Samples were taken from high-touch surfaces e.g. bed edge, touch screen monitors, medication trolley, washbasins.

In addition to the surveillance activities conducted as part of the epidemiological investigation, routine environmental microbiological surveillance for ‘alert microorganisms’ in the ICU was carried out monthly, in accordance with the internal protocols.

#### Microbiological identification and antimicrobial resistance

Species identification and minimum inhibitory concentrations (MIC) were determined using VITEK2 (bioMérieux SA). Carbapenemase production was assessed with an immunochromatographic test (NG-Test CARBA 5). Susceptibility to cefiderocol was assessed using both agar disc diffusion and broth microdilution, while aztreonam/avibactam susceptibility was evaluated with Strip-Test (Liofilchem, Italy). MICs were interpreted according to the European Committee on Antimicrobial Susceptibility Testing (EUCAST) 2025 breakpoints [11].

#### Additional typing method for outbreak investigation

To reconstruct the real epidemic expansion, a rapid in-house procedure, hereafter referred to as NanoTyping, was employed. NanoTyping is based on the amplification of target genes and the sequencing of the resulting amplicons using Oxford Nanopore Technologies (ONT).

Briefly, colonies from freshly cultured strains were resuspended in 200 µL of water and boiled at 99 °C for 10 min. Two microlitres of boiled lysates were used as a template in a 50 µL PCR reaction using OneTaq Quick-Load 2X Master Mix (New England Biolabs). Primers targeted the multilocus sequence typing (MLST) genes, *wzi* gene, *bla*_NDM_, *bla*_KPC_ and *bla*_OXA-48_ carbapenemase genes are provided in Supplementary Material S1 - NanoTyping primers, PCR conditions and references [12-14]. PCR products were purified with GeneMATRIX PCR/DNA CLEAN (EURX, Poland) and sequenced by ONT Rapid Barcoding Kit 96 on R10.4.1 flow cells. The resulting FASTQ files were analysed using the Epi2Me platform with reference sequences (listed in Supplementary Material S1 - NanoTyping primers, PCR conditions and references) using an amplicon workflow as recommended by Epi2Me [15]. Consensus sequences from the amplicons were uploaded to the Institute Pasteur *Klebsiella pneumoniae* MLST webpage (https://bigsdb.pasteur.fr/klebsiella). The first strain isolated was included in the analysis.

### Whole genome sequencing and bioinformatic analysis

The first NDM-producing isolate identified from each patient was selected for whole genome sequencing (WGS) to enable complete plasmid reconstruction and detailed epidemiological analysis. Six prototypical strains were sequenced with the ONT platform as previously described [16]. Briefly, genomic DNA for long-read sequencing was extracted using the Monarch HMW DNA Extraction Kit for Tissue (New England Biolabs). The DNA was used for ONT sequencing with basecalling performed in high accuracy mode.

All the ST147 strains were sequenced with Illumina method. Genomic DNA was extracted from isolates using a GeneMATRIX Bacterial and Yeast Genomic DNA Purification Kit (EURX). Libraries were constructed with the VAHTS Universal Plus DNA Library Prep Kit for Illumina (VAHTS) according to the manufacturer’s instructions. Sequencing was conducted on an NovaSeq 6000 system (Illumina) in paired-end 150 bp (PE150) mode, using the NovaSeq 6000 S4 Reagent Kit. Genome assemblies of both Illumina-only sequencing and hybrid ONT/Illumina outputs were generated with Unicycler 0.5.1 [17] to obtain fully circularised plasmid and chromosomal sequences. Assemblies were annotated with Prokka 1.14.6 [18] and aligned using Snippy 4.6.0 [19]. Phylogenetic trees were constructed with IQ-TREE 2.4.0 [20] and metadata were added with Microreact 274 web tool [21]. Antimicrobial resistance genes (ARGs) and plasmids replicons were identified with StarAMR 0.11.0 [22]. The ARGs and virulence genes were identified with Pathogenwatch 23.3.0–0 [23]. Moreover, genomes of previously described outbreaks [24] or single isolate [25] of co-producing NDM and OXA-48 *K. pneumoniae* isolated in Italy were downloaded from the National Centre for Biotechnology Information. The downloaded sequences were analysed with the same workflow previously mentioned. Figures were edited with Inkscape 1.3.2.

## Results

Contact tracing was initiated following the identification of the five patients colonised with NDM- and OXA-48 co-producing *K. pneumoniae* in the S-ICU (Patients 1, 2, 3, 5 and 6) between 17 February and 6 March 2025. Four patients (Patients 1, 3, 5 and 6) were transferred to the adjacent ICU, where isolation measures could be applied, while Patient 2 was placed in isolation in the T5 ward (W-T5), as they were originally admitted to that ward and had been transferred to the S-ICU only temporarily because of clinical complications. Patient 1 died shortly after being transferred because of unrelated causes.

### Epidemiological investigation

Starting 7 March, active contact surveillance was carried out in accordance with the recommendations provided by the infectious disease specialist of the IPC committee. A total of 30 patients were included in the surveillance. Six patients were still hospitalised in the S-ICU, while the remaining 24 had been transferred to other wards.

During the period of enhanced IPC measures, active surveillance was implemented for all patients admitted to the S-ICU and for the contacts of confirmed cases who had previously been hospitalised in that unit. Active surveillance was concluded on 25 March, when no further positive swabs were detected among the patients under surveillance, 7 days after the last contact with a positive patient.

### Case characteristics

**Figure 1:**
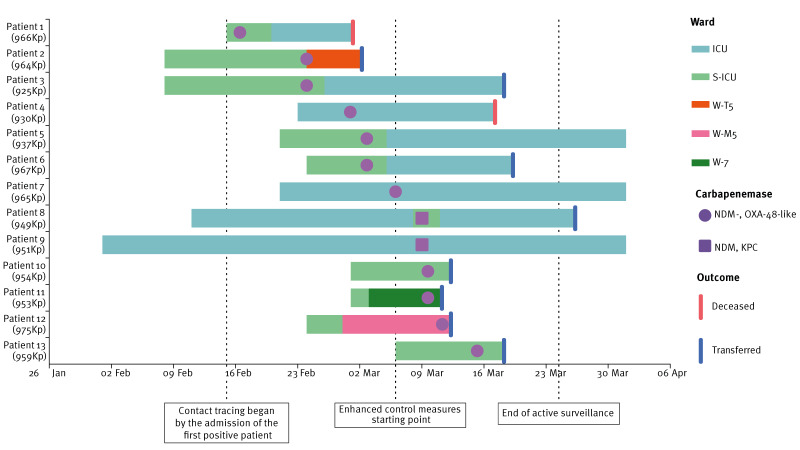
Timeline of hospitalisation, positive cultures and control measures during a hospital outbreak of NDM-producing *Klebsiella pneumoniae*, Rome, Italy, 1 February–31 March 2025 (n = 13 patients)

ICU: intensive care unit; S-ICU: sub-intensive care unit; W: ward.

As a result of the surveillance programme, five additional cases of NDM-producing *K. pneumoniae* were identified from 7 to 15 March, four of which also co-produced OXA-48-like carbapenemase (Figure 1). Three of these strains were isolated in the S-ICU (Patients 8, 10 and 13), and two in other wards (W-7 and W-M5, Patients 11 and 12 respectively), where two other patients previously admitted to the S-ICU had been transferred before the initiation of the active surveillance. Furthermore, three additional cases of NDM-producing *K. pneumoniae* colonisation were identified in the ICU through analysis of active surveillance records (Patients 4, 7 and 9) (Figure 1).

Horizontal timelines show hospital stays of 13 patients with carbapenemase-producing Klebsiella pneumoniae from February to March 2025, colour-coded by ward and carbapenemase type. Dotted lines mark key outbreak control milestones, and symbols indicate patient outcomes (death or transfer).

A total of 13 patients tested positive for NDM-producing *K. pneumoniae* during the study period, four females with median age 63.5 years (range: 54–77), and nine males with median age 61.3 years (range: 20–90). According to our case definition, 12 cases were classified as hospital-acquired, as positivity was detected more than 48 h after hospital admission, whereas only one case was considered imported, having tested positive within 48 h of admission. Eleven patients co-produced NDM and OXA-48-like carbapenemases. In one case, a secondary BSI caused by *K. pneumoniae* co-producing NDM and OXA-48 was diagnosed (Patient 3) and successfully treated with a combination of aztreonam and ceftazidime/avibactam.

### Microbiological investigation

#### Sample sources

Thirteen clinical isolates and one environmental isolate were included in the molecular investigation. Nine strains were isolated from rectal swab samples, while in four patients, they were isolated from lower respiratory tract specimens, including tracheobronchial aspirate or bronchoalveolar lavage (Table).

**Table:** NanoTyping results and antibiotic susceptibility of *Klebsiella pneumoniae* isolates from patients (n = 13) and the environment (n = 1) of a hospital outbreak, Rome, Italy, 1 February–31 March 2025

Strain ID	Patient	Ward	Source	NanoTyping results					Minimum inhibitory concentrations											
				ST	*wzi*	NDM	OXA	KPC	AZA	CAZ	CIP	CN	COL	C/T	CZA	FDC	FDC DISC	I/R	MEM	M/V
966Kp	1	S-ICU	RS	147	104	5	48	Nd	0.19 (S)	≥ 64 (R)	≥ 4 (R)	≥ 16 (R)	0,5 (S)	≥ 32 (R)	≥ 16 (R)	1 (S)	2.1 (ATU)	≥ 16 (R)	≥ 16 (R)	≥ 64 (R)
964Kp	2	S-ICU	TBA	147	420	1	48	Nd	0.25 (S)	≥ 64 (R)	≥ 4 (R)	≥ 16 (R)	0,5 (S)	≥ 32 (R)	≥ 16 (R)	1 (S)	2.1 (ATU)	≥ 16 (R)	≥ 16 (R)	≥ 64 (R)
925Kp	3	S-ICU	BAL	147	420	1	48	Nd	0.125 (S)	≥ 64 (R)	≥ 4 (R)	≥ 16 (R)	0,5 (S)	≥ 32 (R)	≥ 16 (R)	1 (S)	2.1 (ATU)	≥ 16 (R)	≥ 16 (R)	≥ 64 (R)
930Kp	4	ICU	RS	147	420	1	48	Nd	0.25 (S)	≥ 64 (R)	≥ 4 (R)	≥ 16 (R)	0,5 (S)	≥ 32 (R)	≥ 16 (R)	2 (S)	2.1 (ATU)	≥ 16 (R)	≥ 16 (R)	≥ 64 (R)
937Kp	5	S-ICU	BAL	147	420	1	48	Nd	0.38 (S)	≥ 64 (R)	≥ 4 (R)	≥ 16 (R)	0,5 (S)	≥ 32 (R)	≥ 16 (R)	2 (S)	2.1 (ATU)	≥ 16 (R)	≥ 16 (R)	≥ 64 (R)
967Kp	6	S-ICU	RS	147	420	1	48	Nd	0.25 (S)	≥ 64 (R)	≥ 4 (R)	≥ 16 (R)	0,5 (S)	≥ 32 (R)	≥ 16 (R)	2 (S)	2.1 (ATU)	≥ 16 (R)	≥ 16 (R)	≥ 64 (R)
965Kp	7	ICU	TBA	147	420	1	48	Nd	0.38 (S)	≥ 64 (R)	≥ 4 (R)	≥ 16 (R)	8 (R)	≥ 32 (R)	≥ 16 (R)	1 (S)	2.2 (ATU)	≥ 16 (R)	≥ 16 (R)	≥ 64 (R)
949Kp	8	S-ICU	RS	101	137	5	Nd	3	0.38 (S)	≥ 64 (R)	≥ 4 (R)	≥ 16 (R)	4 (R)	≥ 32 (R)	≥ 16 (R)	4 (R)	0.9 (R)	≥ 16 (R)	≥ 16 (R)	≥ 64 (R)
951Kp	9	ICU	RS	512	154	5	Nd	3	0.19 (S)	≥ 64 (R)	≥ 4 (R)	2 (S)	8 (R)	≥ 32 (R)	≥ 16 (R)	2 (S)	1.8 (R)	≥ 16 (R)	≥ 16 (R)	≥ 64 (R)
954Kp	10	S-ICU	RS	147	420	1	48	Nd	0.5 (S)	≥ 64 (R)	≥ 4 (R)	≥ 16 (R)	≥ 8 (R)	≥ 32 (R)	≥ 16 (R)	1 (S)	2 (ATU)	≥ 16 (R)	≥ 16 (R)	≥ 64 (R)
953Kp	11	W-7	RS	147	420	1	48	Nd	0.25 (S)	≥ 64 (R)	≥ 4 (R)	≥ 16 (R)	0,5 (S)	≥ 32 (R)	≥ 16 (R)	2 (S)	2 (ATU)	≥ 16 (R)	≥ 16 (R)	≥ 64 (R)
975Kp	12	W-M5	RS	147	420	1	48	Nd	0.25 (S)	≥ 64 (R)	≥ 4 (R)	≥ 16 (R)	0,5 (S)	≥ 32 (R)	≥ 16 (R)	2 (S)	2.1 (ATU)	≥ 16 (R)	≥ 16 (R)	≥ 64 (R)
959Kp	13	S-ICU	RS	147	420	1	48	Nd	0.38 (S)	≥ 64 (R)	≥ 4 (R)	≥ 16 (R)	0,5 (S)	≥ 32 (R)	≥ 16 (R)	1 (S)	2 (ATU)	≥ 16 (R)	≥ 16 (R)	≥ 64 (R)
AMB201Kp	Nd	ICU	ENV	147	420	1	48	Nd	0.19 (S)	≥ 64 (R)	≥ 4 (R)	≥ 16 (R)	0,5 (S)	≥ 32 (R)	≥ 16 (R)	1 (S)	2.2 (ATU)	≥ 16 (R)	≥ 16 (R)	≥ 64 (R)

ATU: area of technical uncertainty; AZA: aztreonam/avibactam; BAL: bronchoalveolar lavage; CAZ: ceftazidime; CIP: ciprofloxacin; CN: gentamycin; COL: colistin; CZA: ceftazidime/avibactam; C/T: ceftolozane/tazobactam; FDC: cefiderocol broth microdiluition; FDC DISC: cefiderocol agar disc diffusion (in mm); FEP: cefepime; FOS: fosfomycin; ICU: intensive care unit; I/R: imipenem cilastatin relebactam; MEM: meropenem; M/V: meropenem/vaborbactam; Nd: not detected; P/T: piperacillin/tazobactam; RS: rectal swab; R: resistant; S: susceptible; ST: sequence type; S-ICU: sub-intensive care unit; TBA: tracheobronchial aspirate.

NanoTyping results are shown as sequence type and allelic profile of the target genes (multilocus sequence typing (MLST) genes, *wzi* gene, *bla*_NDM_, *bla*_KPC_ and *bla*_OXA-48_ genes), where not detected are indicated as Nd. Minimum inhibitory concentration (MIC) of the most relevant antibiotics tested are shown; MIC interpretation is based on the European Committee on Antimicrobial Susceptibility Testing (EUCAST) 2025 criteria [11].

Environmental samples of 21 different high-touch surfaces were taken in the S-ICU on 18 March. Seven contaminated surfaces were detected, but none were contaminated by NDM-producing *K. pneumoniae* strains. In contrast, routine environmental sampling of 40 high-touch surfaces in the ICU revealed seven contaminated sites, including one positive for NDM/OXA-48-like-producing *K. pneumoniae* on a bed edge (strain AMB201Kp). Supplementary Table S2 presents the detailed results of environmental sampling to facilitate the identification of contaminated areas. The environmental isolate was included in the molecular and genomic analysis, resulting in a total of 14 NDM-producing strains.

#### Microbiological and molecular typing

All the isolates were resistant to all antibiotic classes tested except for aztreonam/avibactam, cefiderocol, colistin and, only in isolate 951Kp, gentamicin. While all isolates were susceptible to aztreonam/avibactam, not all showed susceptibility to cefiderocol and colistin. Notably, isolate 949Kp was susceptible only to aztreonam-avibactam, proving resistant to all the other tested antibiotic compounds, including cefiderocol (Table).

Based on NanoTyping results, 10 patient-derived strains and the environmental isolate (AMB201Kp) were assigned to ST147 and displayed the *wzi*420 allele by *wzi* typing (Table) [13]. These 11 strains carried both *bla*_NDM-1_ and *bla*_OXA-48_ carbapenemase genes and were considered as potentially associated with the outbreak under investigation. In contrast, the isolate 966Kp also assigned to ST147 harboured the *wzi*104 allele and carried the *bla*_NDM-5_ and *bla*_OXA-48_ genes. Two additional *K. pneumoniae* isolates, 949Kp and 951Kp, co-producing NDM-5 and KPC-3, were isolated respectively in the S-ICU and ICU and were assigned to ST101 and ST512 (Table).

### Genomic characterisation

**Figure 2:**
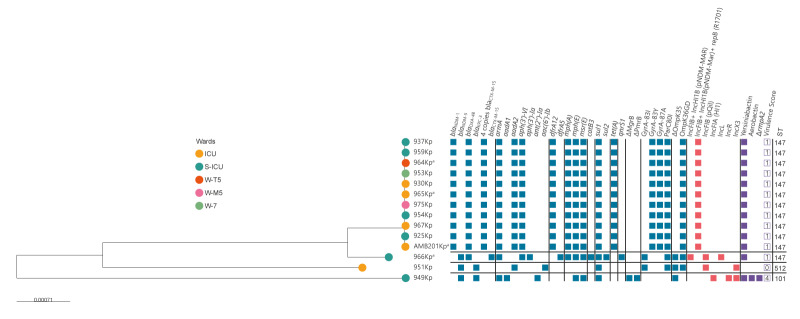
Phylogenetic maximum likelihood SNP-based tree of NDM-producing *Klebsiella pneumoniae* strains isolated from patients (n = 13) and the environment (n = 1) during a hospital outbreak, Rome, Italy, February–March 2025

ICU: intensive care unit; S-ICU: sub-intensive care unit; ST: sequence type; W: ward.

^a^ Strains subjected to hybrid assembly.

A phylogenetic tree with the 14 isolates under investigation was reconstructed. The phylogenetic analysis revealed one major clade consisting of 11 strains originating from multiple wards. All these strains harboured *bla*_NDM-1_ and *bla*_OXA-48_ carbapenemase genes as well as the extended spectrum beta-lactamase *bla*_CTX-M-15_. Hybrid assembly results showed as the ST147 outbreak related strains harboured the *bla*_NDM-1_ gene located on a derivative of pNDM-MAR plasmid, also positive for the replicon repB_R1701 (Figure 2). Instead, the *bla*_OXA-48_ and four copies of *bla*_CTX-M-15_ genes were integrated into the chromosome, along to the virulence determinant *Ybt* locus (Yersiniabactin). Three additional branches were identified in the phylogenetic tree (Figure 2). One branch was represented by the hybrid-assembled genome of the isolate 966Kp, also ST147 but with a different resistance genes and plasmids profile, and lacking the chromosomal integrations observed in the other outbreak-related ST147 strains. Another branch was represented by isolate 951Kp belonging to ST512, harbouring a *bla*_KPC-3_ carbapenemase gene on a pKpQIL plasmid and the *bla*_NDM-5_ gene on an IncX3 plasmid. The last branch is represented by 949Kp ST101 strain that harbour *bla*_KPC-3_ gene on an IncFIA (HI1) plasmid and the *bla*_NDM-5_ gene on an IncX3 plasmid. This isolate presents chromosomal absence of *mrgB* and *pmrB* genes. Moreover, it presents virulence determinants such as *Ybt* and *Iro* (Aerobactin) loci (Figure 2). Isolates 949Kp and 951Kp were sequenced only with the ONT method. Additional resistance determinants against several antibiotic classes and plasmids as *Col(BS512)*, *Col440II*, and *ColRNAI* were also detected. A complete list of antibiotic resistance genes tested in each isolate is provided in Supplementary Table S3.

Each branch point is colour-coded according to the isolation ward, as indicated in the legend. The most relevant detected antimicrobial resistance genes, plasmid replicons and virulence genes are displayed. Virulence scores and sequence types are also reported. A complete gene list is added as Supplementary Table S3.

### Comparison of NDM and OXA-48 co-producing *Klebsiella pneumoniae* strains in Italy

**Figure 3:**
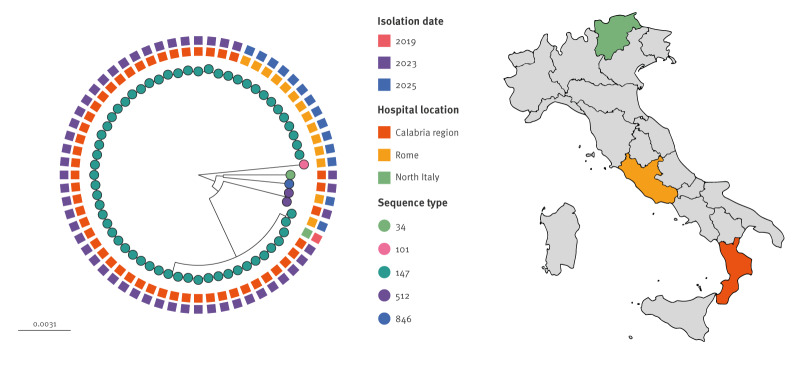
Phylogenetic tree of all non-duplicate NDM-producing *Klebsiella pneumoniae* strains characterised in Italy (n = 64), including NDM-producing strains in our study (n = 14), Rome, Italy, February–March 2025

We compared the genomes sequenced here, with the only available genomic data of Italian strains co-harbouring *bla*_NDM_ and *bla*_OXA-48_ genes: strains of an Italian outbreak in Calabria (2023), a southern Italian region, and one strain isolated in northern Italy [24,25]. A phylogenetic tree was constructed (Figure 3). Except for isolate 966Kp and the strain isolated in northern Italy in 2019, which cluster on a separate branch, all other ST147 strains from this outbreak within the same branch as the Calabrian isolates.

The isolation date, hospital location (region of isolation), and sequence type are annotated according to the legend. For geographical context, a map of Italy is included, with regions of strain origin highlighted using corresponding colours from the legend.

## Outbreak control measures

In response to reporting of the first five cases, the hospital's IPC committee was promptly convened, and further containment actions were implemented, including the suspension of non-urgent patient transfers and active surveillance of contacts. Essential clinical transfers for unavoidable needs continued; the majority of transfers occurred before 6 March, i.e. the start of the enhanced measures, with only Patient 8 transferred during this period. Supplementary Material S4 provides a summary of control measures.

Strict adherence to IPC measures was required of all healthcare personnel, with close supervision and systematic documentation of compliance. Implemented measures included hand hygiene recommendations, thorough cleaning and disinfection of the environment and infrastructure, as well as sanitisation of electro-medical equipment and frequently touched surfaces in both patient and staff areas. Healthcare workers were assigned exclusively to patients in isolation to avoid cross-contamination. Active surveillance of contacts with rectal swabs was initiated on 7 March and continued until no further positive swabs were detected among the patients under surveillance, on 25 March.

## Discussion

We described the epidemiological and microbiological investigation and the containment measures adopted in the first documented hospital outbreak sustained by a *K. pneumoniae* ST147/wzi420/NDM-1/OXA-48 clone in Rome, Italy. NDM- and OXA-48–co-producing *K. pneumoniae* strains were uncommon in our hospital before this event and occurred only as isolated, sporadic cases, in contrast to the outbreak we documented. The outbreak rapidly spread between two different wards (S-ICU and ICU) and involved a total of 10 patients over 4 weeks, underscoring the importance of rapid detection, coordinated surveillance and timely implementation of IPC measures against MDRO. Indeed, while the clone responsible for the outbreak had a low predicted virulence score, as only one of the 10 affected patients subsequently developed a BSI, its extensive antibiotic resistance profile is concerning, making it imperative to contain the transmission.

This outbreak exemplifies the growing complexity of containing MDROs within modern hospital systems. A key public health concern raised by this investigation is the capacity for cross-ward transmission, particularly in large tertiary care hospitals with interconnected clinical pathways. Five of the identified cases were detected after patient transfer from the S-ICU to the ICU or other wards, before the implementation of active surveillance. This suggests that the delay between colonisation, strain detection and isolation, in addition to the technical turnaround time of rectal swab testing, may have facilitated silent dissemination across wards. This is consistent with previous findings highlighting that patient movement within and between facilities is a major driver of MDRO spread [26]. In addition, the outbreak spread beyond its initial location despite separation of staff and equipment between units. This suggests potential breaches in protocol or unrecognised transmission routes. High patient acuity, frequent need for urgent interventions, and staff shortages often compromise strict adherence to contact precautions practices. Studies have shown compliance with hand hygiene and gown/glove protocols to be as low as 40% during night shifts or emergencies [27]. Furthermore, the complexity of patients in ICUs further constraints to traditional ‘one patient–one room’ isolation models, particularly in settings with limited space [28]. Nevertheless, reinforced adherence to IPC protocols, including hand hygiene, dedicated equipment and enhanced environmental cleaning remains crucial, as they are the most cost-effective way to prevent cross-contamination [29].

We isolated the same outbreak-causing strains in both patients and hospital environment samples, which highlights the importance of the environment in dissemination of such strains [30]. Studies have reported *Klebsiella* spp. to survive between 90 and 600 days on inanimate porous surfaces [31]. These suggest the need to reinforce environmental cleaning measures and that routine environmental surveillance, especially during outbreak investigations, can help identify hidden reservoirs and guide targeted cleaning [32].

Despite possible breaches in IPC measures that might have occurred, the early detection and containment of this outbreak were facilitated by the screening of ICU and S-ICU patients with rectal swabs implemented in the hospital, and by the routine active surveillance protocols in place in the ICU since 2016; these actions allowed the identification of additional cases in patients not originally included in the surveillance of contacts. The risk of secondary infection reported in our study (1/10 cases) is consistent with previous data, suggesting the relevance of screening in high-risk patient populations [33]. The rapid implementation of contact tracing and cohorting contributed to halting further spread and is an important response to detection of high-risk clones.

The molecular typing (NanoTyping) approach proved to be a powerful and rapid frontline tool for real-time outbreak investigation and was subsequently confirmed by WGS. NanoTyping results confirmed the presence of multiple distinct clones among the analysed strains. Notably, 11 ST147/wzi420/NDM-1/OXA-48 strains were identified as the true outbreak-related clone. These isolates exhibited genotypic features rarely reported in Italy and closely related only to a previous outbreak in a southern Italian region [24]. This strategy may enhance local outbreak detection capabilities and accelerate IPC responses, particularly in hight MDRO prevalence settings where conventional WGS resources are limited or turnaround times are prolonged.

The WGS results confirmed relatedness among the 11 *K. pneumoniae* ST147, co-harbouring the *bla*_NDM-1_ and *bla*_OXA-48_ genes, obtained from 10 patients and the ICU environmental isolate AMB201Kp. The strain 966Kp was confirmed not to be part of the outbreak although it belonged to ST147. The outbreak-related strains exhibited an unusual genomic content that distinguished them from 966Kp. Isolates 951Kp and 949Kp, belonging to ST512 and ST101 respectively, were spurious NDM-producing strains not related to the outbreak. However, strain 949Kp displayed a concerning convergent phenotype, carrying both resistance and virulence determinants. Although this was not an outbreak-causing strain, it highlights the well-documented phenomenon of evolutionary convergence between virulence and antimicrobial resistance traits [34].

A comparison with other Italian genomes of closely related isolates revealed that, despite a 2-year gap from the outbreaks reported in Calabria, a virtually identical ST147 clone, differing by around only 20 SNPs from the most closely related strains identified in this study, was responsible for the outbreak described in Rome. Although this ST147 clone is currently rare in Italy and has previously been reported only in the Calabria region, its expansion across different regions since 2023, its high level of antimicrobial resistance, and its ability to cause rapid hospital outbreaks are concerning traits of this emerging clone. The characteristic chromosomal integration of resistance determinants in this clone may confer enhanced stability of the resistance phenotype and pose additional challenges for containment [35]. At the European level, clones harbouring both *bla*_NDM_ and *bla*_OXA-48_ are increasingly being reported [6,7], highlighting how several distinct STs show convergent evolutionary trajectories towards this gene combination, suggesting a possible selective advantage.

Our study has several limitations. Firstly, we were unable to clearly identify the primary case of the outbreak. The isolate of the first patient, which was imported from outside the hospital and co-produced NDM and OXA-48 carbapenemases (966Kp), was not related to the outbreak. Furthermore, subsequent positive patients had previous negative rectal swabs, suggesting that the colonisation likely occurred within the hospital. However, although we were not able to identify the primary case, we are confident that a true outbreak occurred in these wards, as the ST147 NDM- and OXA-48–co-producing *Klebsiella pneumoniae* clone had never previously been identified in these units. Moreover, NDM- and OXA-48–co-producing *K. pneumoniae* strains were uncommon in our hospital before this event and occurred only as isolated, sporadic cases, in contrast to the outbreak we documented. Secondly, this study focused only on patients admitted to the ICU and S-ICU, where routine surveillance is performed upon admission and on a weekly basis, allowing the prompt detection of colonisation. However, this approach did not include patients from other wards, nor patients who were discharged before the initiation of the active surveillance, potentially underestimating the extent of transmission within the hospital. Finally, NDM- and OXA-48–co-producing *K. pneumoniae* strains previously sporadically identified in other wards are no longer available for further analysis.

## Conclusion

This outbreak highlights the ongoing risk posed by a high-risk *K. pneumoniae* clone co-producing NDM and OXA-48 carbapenemases. Screening of newly admitted patients, the implementation of active surveillance of contacts, and the integration of rapid molecular typing into routine surveillance proved essential for timely outbreak detection and containment. Given the extensive resistance profile and epidemic potential of the ST147 clone involved, sustained molecular surveillance, including environmental surveillance, and reinforced IPC measures are crucial to prevent further spread. Strengthening screening and genotyping capacity should be a priority in healthcare settings facing high burdens of MDRO.

## Data Availability

Sequenced genomes are accessible as SRA of both, Illumina and Oxford Nanopore Technologies sequencing on BioProject ID: PRJNA1260529.

## Supplementary Data

354000 Supplementary Material
