# Supplementary material for: Hospital outbreak sustained by Klebsiella pneumoniae sequence type 147 co-producing NDM-1 and OXA-48, Rome, Italy, February to March 2025: molecular tracing and control measures
Source: Euro Surveill. 2026 Mar 12;31(10):2500457. doi: 10.2807/1560-7917.ES.2026.31.10.2500457 (PMC13074120; doi:10.2807/1560-7917.ES.2026.31.10.2500457)
Supplement: Supplementary Material [file 25-00457_ROSSO_Supplement.pdf]

This supplementary material is hosted by Eurosurveillance as supporting information alongside the article “Hospital outbreak sustained by *Klebsiella pneumoniae* sequence type 147 co-producing NDM-1 and OXA-48, Rome, Italy, February to March 2025: molecular tracing and control measures”, on behalf of the authors, who remain responsible for the accuracy and appropriateness of the content. The same standards for ethics, copyright, attributions and permissions as for the article apply. Supplements are not edited by Eurosurveillance and the journal is not responsible for the maintenance of any links or email addresses provided therein.

Supplementary Material S1. NanoTyping primers, PCR conditions and references

Nanotyping primers

ID	Fw	Rv	REF	Bp
**rpoB**	GTTTTCCCAGTCACGACGTTGTA<br>GGCGAAATGGCWGAGAACCA	TTGTGAGCGGATAACAATTTC<br>GAGTCTTCGAAGTTGTAACC	[1]	1119
**gapA**	GTTTTCCCAGTCACGACGTTGTA<br>TGAAATATGACTCCACTCACGG	TTGTGAGCGGATAACAATTTC<br>CTTCAGAAGCGGCTTTGATGGCTT	[1]	706
**mdh**	GTTTTCCCAGTCACGACGTTGTA<br>CCCAACTCGCTTCAGGTTCAG	TTGTGAGCGGATAACAATTTC<br>CCGTTTTTCCCCAGCAGCAG	[1]	800
**pgi**	GTTTTCCCAGTCACGACGTTGTA<br>GAGAAAAACCTGCCTGTACTGCTGGC	TTGTGAGCGGATAACAATTTC<br>CGCGCCACGCTTTATAGCGGTTAAT	[1]	709
**phoE**	GTTTTCCCAGTCACGACGTTGTA<br>ACCTACCGCAACACCGACTTCTTCGG	TTGTGAGCGGATAACAATTTC<br>TGATCAGAACTGGTAGGTGAT	[1]	646
**infB**	GTTTTCCCAGTCACGACGTTGTA<br>CTCGCTGCTGGACTATATTCG	TTGTGAGCGGATAACAATTTC<br>CGCTTTCAGCTCAAGAACTTC	[1]	506
**tonB**	GTTTTCCCAGTCACGACGTTGTA<br>CTTTATACCTCGGTACATCAGGTT	TTGTGAGCGGATAACAATTTC<br>ATTCGCCGGCTGRGCRGAGAG	[1]	583
**wzi_long**	GTTTTCCCAGTCACGACGTTGTA<br>GTGCCGCGAGCGCTTTCTATCTTGGTATTCC	TTGTGAGCGGATAACAATTTC<br>GAGAGCCACTGGTTCCAGAAYTTSACCGC	[2]	624
**NDM_long**	GTTTTCCCAGTCACGACGTTGTA<br>GGCGAAATATGGAATTGCCCAATATTATGCACCC	TTGTGAGCGGATAACAATTTC<br>TCAGCGCAGCTTGTCGGC	in house	875
**OXA-48**	TATATTGCATTAAGCAAGGG	CACACAAATACGCGCTAACC	[3]	800
**KPC_long**	GTTTTCCCAGTCACGACGTTGTA<br>GGCGAAATATGTCACTGTATCGCCGTCT	TTGTGAGCGGATAACAATTTC<br>TTTTCAGAGCCTTACTGCCC	In house	922

Colonies from freshly grown strains on overnight UTI Chromatic agar plates (Liofilchem, Italy) were resuspended in 1 mL of water and boiled at 99°C for 10 minutes. Two microliters of boiled lysates were used as template in a 50 µL PCR reaction using OneTaq Quick-Load 2X Master Mix (NEB, USA).

PCR conditions:

- **1 cycle:**
  - **94° for 10 min**
- **35 cycles:**
  - **94° for 30 s**
  - **50°C for 1 min**
  - **72° for 30 s**
- **1 cycle:**
  - **72° for 5 min**

Reference sequences for EPI2ME wf-amplicon

ID	Sequence
**rpoB**	ACCCACTACGGTCGCGTATGTCCGATCGAAACGCCTGAAGGTCCGAACATCGGTCTGATT<br>AACTCCCTGTCCGTGTACGCGCAGACCAACGAATATGGCTTCCTTGAGACGCCGTATCGT<br>AAAGTGACCAACGGTGTGGTTACTGACGAAATTCACTACCTGTCTGCTATCGAAGAAGGC<br>AACTACGTTATCGCTCAGGCGAACTCCAACCTGGATGAAAACGGCCACTTCGTAGAAGAT<br>CTGGTTACCTGCCGTAGCAAAGGCGAATCCAGCTTGTTCAGCCGCGACCAGGTTGACTAC<br>ATGGACGTATCCACCCAGCAGGTGGTATCCGTCGGTGCGTCCCTGATCCCGTTCCTGGAA<br>CACGATGACGCCAACCGTGCATTGATGGGTGCGAACATGCAACGTCAGGCGGTTCCGACT<br>CTGCGCGCTGATAAGCCGCTGGTTGGTACCGGTATGGAACGTGCTGTTGCCGTTGACTCC<br>GGTGTTACTGCCGTGGCTAAA
**gapA**	AACCTGAAGTGGGACGAAGTTGGTGTTGACGTTGTTGCTGAAGCAACCGGTATCTTCCTG<br>ACCGACGAAACCGCTCGTAAACACATCACCGCTGGCGCGAAAAAAGTCGTTCTGACTGGC<br>CCGTCCAAAGACAACACTCCGATGTTCGTTCGCGGCGCTAACTTCGACGCTTACGATGGC<br>CAGGACATCGTTTCCAACGCTTCCTGCACCACTAACTGCCTGGCGCCGCTGGCTAAAGTT<br>ATCAACGACAACTTCGGTATCGTTGAAGGCCTGATGACCACCGTCCACGCTACCACCGCT<br>ACTCAGAAAACCGTTGATGGCCCGTCTCACAAAGACTGGCGCGGCGGCCGCGGCGCAGCT<br>CAGAACATCATCCCGTCCTCTACCGGCGCTGCTAAAGCAGTAGGTAAAGTACTGCCAGAA<br>CTGAACGGCAAACTGACCGGTATGGCGTTC
**mdh**	GGCGCGGATGTAGTGCTGATCTCCGCGGGCGTGGCGCGTAAGCCCGGCATGGATCGTTCC<br>GACCTGTTTAATGTGAATGCGGGTATCGTGAAGAACCTCGTGCAGCAGATTGCCAAAACC

	TGCCCGCAGGCCTGCATCGGCATTATCACCAACCCGGTGAATACCACCGTGGCTATCGCC<br>GCCGAAGTACTGAAAAAAGCCGGCGTGTACGATAAAAACAAACTGTTCGGCGTTACCACG<br>CTGGACATCATCCGTTCCAATACCTTTGTGGCGGAGCTGAAAGGTAAATCGGCAACCGAG<br>GTGGAAGTCCCGGTCATTGGTGGTCACTCCGGGGTCACCATTCTGCCTTTACTGTCGCAG<br>ATCCCCGGCGTCAGCTTTAGCGATCAGGAAATTGCCGACCTGACTAAACGTATTCAGAAC<br>GCCGGTACCGAAGTCGTGGAAGCGAAAGCGGGCGGCGGGTCGGCGACCTTGTCGATG
**pgi**	GAGTCCAACGGTAAGTATGTTGACCGTAACGGCCACGCGGTAGACTACCAGACTGGCCC<br>AATCATCTGGGGGGAGCCGGGCACCAACGGTCAGCACGCGTTCTACCAGCTGATCCACC<br>AGGGCACCAAAATGGTACCGTGCGATTTCATCGCTCCGGCTATCACCCACAACCCGCTGTC<br>TGACCACCATCAGAAACTGCTGTCTAACTTCTTCGCCCAGACCGAGGCCCTGGCCTTTGGTA<br>AATCCCGCGAAGTGGTTGAGCAGGAATATCGCGATCAGGGTAAAGACCCGGCGACCCTGG<br>AGCACGTGGTGCCGTTCAAAGTGTTCGAAGGTAACCGCCCGACTAACTCCATCCTGCTGCGT<br>GAGATCACCCCGTTCAGCCTCGGGGCGCTGATTGCCCTGTACGAGCACAAAATCTTCACCCAGGGCGCG
**phoE**	GTCGGCACCTCGTTAAGCTATGATTTCGGCGGCAGCGACTTCGCCGTCAGCGCAGCCTAC<br>ACCAGCTCCGACCGTACCAACGATCAGAACCTGCTGGCCCGCGGCCAGGGTTCGAAAGCG<br>GAAGCCTGGGCGACCGGCCTGAAATATGACGCCAACAATATCTACCTGGCGACCATGTAC<br>TCTGAAACCCGCAAGATGACCCCGATCAGCGGCGGCTTTGCCAACAAAGCGCAGAACTTT<br>GAAGCGGTGGCGCAGTATCAGTTCGACTTCGGTCTGCGTCCGTCCCTCGGCTATGTGCTG<br>TCGAAAGGGAAGGATATCGAAGGGGTGGGGAGTGAAGATCTGGTTAACTACATCGACGTG<br>GGCCTGACCTACTACTTCAACAAAAACATGAACGCCTTCGTGGATTACAAAATCAACCAG
**infB**	GGCATGATCACCTTCCTGGATACCCCGGGCCACGCCGCGTTTACCTCCATGCGTGCTCGT<br>GGCGCGCAGGCGACGGATATCGTGGTTCTGGTGGTGGCGGCAGACGACGGCGTGATGCCG<br>CAGACTATCGAAGCTATCCAGCACGCTAAAGCGGCGCAGGTACCGGTGGTAGTGGCGGTG<br>AACAAGATCGATAAGCCAGAAGCCGATCCGGATCGCGTGAAGAACGAACTGTCCCAGTAC<br>GGCATCCTGCCGGAAGAGTGGGGCGGCGAGAGCCAGTTTGTCCACGTTTCCGCGAAAGCG<br>GGTACCGGCATCGACGAC
**tonB**	ATGGTGGCGCCGGCCGATCTTGAGCCGCCTCCGGCGGCGCAGCCTGTCGTGGAGCCCGTT<br>GTTGAACCCGAACCTGAGCCGGAGCCAGAGGTAGCGCCTGAACCGCCGAAAGAGGCGTCG<br>GTGGTGATCCATAAACCGGAACCTAAGCCGAAGCCCAAACCTAAACCCAAGCCTAAGCCG<br>GAGAAAAAGGTTGAACAGCCGAAGCGGGAAGTGAAGCCGGCAGCAGAGCCGCGTCCGGCC<br>TCGCCGTTTGAAAACAACAATACGGCGCCGGCGCGTACAGCGCCAAGCACCTCGACCGCA<br>GCGGCTAAACCCACCGTTACTGCTCCAAGCGGCCCGCGGGCGATCAGCCGCGTTCAGCCG<br>TCCTATCCGGCGCGCGCTCAGGCGCTGCGCATTGAAGGGACGGTACGGGTGAAG
**wzi**	ATGATAAAAATTGCGCGCATTGCCGTGACGCTGGGCTTGCTTTCCTCACTGGGAGTCCAGGCTT<br>ACGCGGCCGGGTTAGTAGTAAATGATAATGATCTCCGTAACGACCTCGCCTGGCTTTCCGATCG<br>CGGGATCATCCATCTGAGCTTGTCGACCTGGCCGCTGAGCCAGGAAGAGATCGCCCGGGCG<br>CTAAAAAAGGCCAAACCTTCCTATTCCTCTGAGCAGGTAGTTCTGGCTCGTATCAATCAGCGACT<br>GTCAGCGTTAAAAGCCGATTTCCGGGTCACCGGCTATACCTCGACCGACCAGCCGGGCACCC<br>CGCAGGGGTTCGGTCAGACGCAGCCGGCAGATAACTCGTTAGGCCTGGCGTTCAACAACAGCG

	GCGAGTGGTGGGATGTCCATCTGCAGGGCAATGTCGAAGGGGGAGAGCGGATCAGCAACGGCT<br>CGCGC
**NDM**	ATGGAATTGCCCAATATTATGCACCCGGTCGCGAAGCTGAGCACCGCATTAGCCGCTGCATTGAT<br>GCTGAGCGGGTGCATGCCCGGTGAAATCCGCCCGACGATTGGCCAGCAAATGGAAACTGGCGA<br>CCAACGGTTTGGCGATCTGGTTTTCCGCCAGCTCGCACCGAATGTCTGGCAGCACACTTCCTATCT<br>CGACATGCCGGGTTTCGGGGCAGTCGCTTCCAACGGTTTGATCGTCAGGGATGGCGGCCGCGTG<br>CTGGTGGTCGATACCGCCTGGACCGATGACCAGACCGCCCAGATCCTCAACTGGATCAAGCAGGA<br>GATCAACCTGCCGGTCGCGCTGGCGGTGGTGACTCACGCGCATCAGGACAAGATGGGCGGTATG<br>GACGCGCTGCATGCGGCGGGGATTGCGACTTATGCCAATGCGTTGTCGAACCAGCTTGCCCCGCAA<br>GAGGGGATGGTTGCGGCGCAACACAGCCTGACTTTCGCCGCCAATGGCTGGGTCGAACCAGCAACC<br>GCGCCCAACTTTGGCCCGCTCAAGGTATTTTACCCCGGCCCCGGCCACACCAGTGACAATATCACCG<br>TTGGGATCGACGGCACCGACATCGCTTTTGGTGGCTGCCTGATCAAGGACAGCAAGGCCAAGTCGCTC<br>GGCAATCTCGGTGATGCCGACACTGAGCACTACGCCGCGTCAGCGCGCGCGTTTGGTGCGGCGTTCC<br>CCAAGGCCAGCATGATCGTGATGAGCCATTCCGCCCCCGATAGCCGCGCCGCAATCACTCATACGGC<br>CCGCATGGCCGACAAGCTGCGCTGA
**OXA-48**	ATGCGTGTATTAGCCTTATCGGCTGTGTTTTTGGTGGCATCGATTATCGGAATGCCTGCGGTAGCAAAGGAATGGCAAGAAAACAAAAGT<br>TGGAATGCTCACTTTACTGAACATAAATCACAGGGCGTAGTTGTGCTCTGGAATGAGAATAAGCAGCAAGGATTTACCAATAATCTTAAA<br>CGGGCGAACCAAGCATTTTTACCCGCATCTACCTTTAAAATTCCCAATAGCTTGATCGCCCTCGATTTGGGCGTGGTTAAGGATGAACAC<br>CAAGTCTTTAAGTGGGATGGACAGACGCGCGATATCGCCACTTGGAATCGCGATCATAATCTAATCACCGCGATGAAATATTCAGTTGTG<br>CCTGTTTATCAAGAATTTGCCCGCCAAATTGGCGAGGCACGTATGAGCAAGATGCTACATGCTTTCGATTATGGTAATGAGGACATTTCG<br>GGCAATGTAGACAGTTTCTGGCTCGACGGTGGTATTCGAATTTCGGCCACGGAGCAAATCAGCTTTTTAAGAAAGCTGTATCACAATAAG<br>TTACACGTATCGGAGCGCAGCCAGCGTATTGTCAAACAAGCCATGCTGACCGAAGCCAATGGTGACTATATTATTCGGGCTAAAACTGGA<br>TACTCGACTAGAATCGAACCTAAGATTGGCTGGTGGGTCGGTTGGGTTGAACTTGATGATAATGTGTGGTTTTTTGCGATGAATATGGAT<br>ATGCCCACATCGGATGGTTTAGGGCTGCGCCAAGCCATCACAAAAGAAGTGCTCAAACAGGAAAAAATTATTCCCTAG
**KPC**	ATGTCACTGTATCGCCGTCTAGTTCTGCTGTCTTGTCTCTCATGGCCGCTGGCTGGCTTTTCTGCCACCGC<br>GCTGACCAACCTCGTCGCGGAACCATTCGCTAAACTCGAACAGGACTTTGGCGGCTCCATCGGTGTGTA<br>CGCGATGGATACCGGCTCAGGCGCAACTGTAAGTTACCGCGCTGAGGAGCGCTTCCCACTGTGCAGCT<br>CATTCAAGGGCTTTCTTGCTGCCGCTGTGCTGGCTCGCAGCCAGCAGCAGGCCGGCTTGCTGGACACAC<br>CCATCCGTTACGGCAAAAATGCGCTGGTTCCGTGGTCACCCATCTCGGAAAAATATCTGACAACAGGCAT<br>GACGGTGGCGGAGCTGTCCGCGGCCGCCGTGCAATACAGTGATAACGCCGCCGCCAATTTGTTGCTGAA<br>GGAGTTGGGCGGCCCGGCCGGGCTGACGGCCTTCATGCGCTCTATCGGCGATACCACGTTCCGTCTGGA<br>CCGCTGGGAGCTGGAGCTGAACTCCGCCATCCCAGGCGATGCGCGCGATACCTCATCGCCGCGCGCC<br>GTGACGGAAAGCTTACAAAAACTGACACTGGGCTCTGCACTGGCTGCGCCGCAGCGGCAGCAGTTTGTT<br>GATTGGCTAAAGGGAAACACGACCGGCAACCACCGCATCCGCGCGGCGGTGCCGGCAGACTGGGCA<br>GTCGGAGACAAAACCGGAACCTGCGGAGTGTATGGCACGGCAAATGACTATGCCGTCGTCTGGCCCAC<br>TGGGCGCGCACCTATTGTGTTGGCCGTCTACACCCGGGCGCCTAACAAGGATGACAAGTACAGCGAGG<br>CCGTCATCGCCGCTGCGGCTAGACTCGCGCTCGAGGGATTGGGCGTCAACGGGCAGTAA

References

1. Institut Pasteur MLST databases and software [Internet]. [cited 2025 Mar 10]. Available from: https://bigsdb.pasteur.fr/klebsiella/primers-used/

2. Brisse S, Passet V, Haugaard AB, Babosan A, Kassis-Chikhani N, Struve C, et al. wzi Gene Sequencing, a Rapid Method for Determination of Capsular Type for Klebsiella Strains. Journal of Clinical Microbiology. 2013 Dec;51(12):4073–8.

3. LCPDb-ARG [Internet]. [cited 2023 Apr 20]. Available from: http://lcpdb.ddlemb.com/args/

Supplementary Material S2. Results of environmental microbiological tests

Results of microbiological tests on samples taken from surfaces in the S-ICU ward (sampling date: 18/03/25)

Sample Code	Collection Point	Outcome
19829S	Bed edge 1	No isolation
19830S	Bed infusion pump buttons 1	No isolation
19831S	Bed edge 2	*Enterococcus faecalis N-MDR*[1]
19832S	Bed monitor 2	No isolation
19833S	Bed edge 3	No isolation
19834S	Bed fan 3	No isolation
19835S	Bed edge 5	*Acinetobacter baumannii XDR*[3]
19836S	Bed fan 5	*Pantoea spp* NA[4]
19837S	Medication trolley bed 5	No isolation
19838S	Bedside table 5	*Klebsiella pneumoniae* XDR[3]
19839S	Bed infusion pump buttons 7	*Enterococcus faecalis N-MDR*[1]
19840S	Bed edge 7	No isolation
19841S	Bedside table 7	No isolation
19842S	Bed monitor 7	No isolation
19843S	Bed edge 15	*Klebsiella pneumoniae* XDR[3], *Acinetobacter baumannii* XDR[3]
19844S	Bedside trolley 15	No isolation
19845S	Blood gas	No isolation
19846S	Bedside washbasin 15	No isolation
19847S	Bed intubation trolley 13	*Enterococcus faecium* MDR[2]
19848S	Medication trolley bed 18	No isolation
19849S	Mouse + keyboard (main PC)	No isolation

[1]Non Multi Drug Resistant, N-MDR; [2] Multi Drug Resistant, MDR; [3] Extensively Drug Resistant, XDR; [4]Not attributable, NA;

Results of microbiological tests on samples taken from surfaces in the ICU ward (sampling date: 10/03/25)

Area	Sample Code	Collection Point	Outcome
Room 1	19809S	Bed edge 1	No isolation
	19810S	Bed monitor 2	No isolation
	19811S	washbasin	No isolation
Room 2	19812S	washbasin	No isolation
	19813S	Bed edge 3	*Klebsiella pneumoniae* NDM, OXA48 XDR[3]
	19814S	Medication trolley	No isolation
Room 3	19815S	Medication trolley bed 5	No isolation
	19816S	Bed monitor 5	No isolation
	19817S	washbasin	No isolation
Room 4	19818S	Bed edge 8	No isolation
	19819S	Bed monitor 8	No isolation
	19820S	washbasin	No isolation
Room 6	19821S	Bed edge 9	*Enterobacter cloacae* XDR[3]
	19822S	Medication trolley bed 9	No isolation
Room8	19823S	Medication trolley bed 12	No isolation
	19824S	Blood gas	No isolation
	19825S	Bed edge 16	No isolation
	19826S	Medication trolley bed 13	No isolation
	19827S	Bed edge 15	No isolation
	19828S	Medication trolley bed 16	No isolation

[1]Non Multi Drug Resistant, N-MDR; [2] Multi Drug Resistant, MDR; [3] Extensively Drug Resistant, XDR.

Results of microbiological tests on samples taken from surfaces in the ICU ward (sampling date: 31/03/25)

Area	Sample Code	Collection Point	Outcome
Room 8	19866S	Medication trolley bed 18	No isolation
	19867S	Blood gas	No isolation
	19868S	Medicine cabinet handles	No isolation
	19869S	Medication trolley bed 15	No isolation
	19870S	Bed monitor 13	No isolation
	19871S	Medication trolley bed 13	No isolation
	19872S	Bed edge 13	No isolation
Room 7	19873S	Bed edge 11	*Klebsiella pneumoniae MDR*[1]
	19874S	Medication trolley bed 11	No isolation
Room 6	19875S	Medication trolley bed 9	No isolation
Room 4	19876S	Bed edge 7	No isolation
	19877S	Washbasin	*Acinetobacter baumannii complex* XDR[3]
Room 3	19878S	Bed monitor 6	No isolation
	19879S	Bed edge 5	*Pseudomonas oleovorans*<br>*Enterococcus faecalis*<br>*Acinetobacter baumannii* XDR[3]
Room 1	19880S	Bed edge 1	No isolation
	19881S	Washbasin	No isolation
	19882S	Medication trolley bed 1	No isolation
	19883S	Medication trolley bed 2	No isolation
Room 2	19884S	Bed monitor 4	*Acinetobacter baumannii* PDR[4]
	19885S	Washbasin	*Acinetobacter baumannii* XDR[3]

[1]Non Multi Drug Resistant, N-MDR; [2] Multi Drug Resistant, MDR; [3] Extensively Drug Resistant, XDR; [4] Pan Drug Resistant, PDR; [5]Not attributable, NA.

Supplementary Table S3: Complete list of antibiotic resistance genes tested in each isolate

ID	949Kp	951Kp	966Kp	964Kp	965Kp	AMB201Kp	925Kp	930Kp	937Kp	953Kp	954Kp	959Kp	967Kp	975Kp
ST	101	512	147	147	147	147	147	147	147	147	147	147	147	147
Ward	S-ICU	ICU	S-ICU	S-ICU	ICU	ICU	S-ICU	ICU	S-ICU	W-7	S-ICU	S-ICU	ICU	W-M5
Source	TR	TR	TR	TBA	TBA	Bordo letto	BAL	TR	BAL	TR	TR	TR	TR	TR
ARR-3			X											
aac(6')-Ib-cr			X											
aph(3')-Ia			X											
aph(3')-VI			X	X	X	X	X	X	X	X	X	X	X	X
armA	X		X	X	X	X	X	X	X	X	X	X	X	X
aadA2		X		X	X	X	X	X	X	X	X	X	X	X
aadA1	X													
ant(2")-Ia	X													
aac(6')-Ib		X												
blaNDM-1				X	X	X	X	X	X	X	X	X	X	X
blaNDM-5	X	X	X											
blaOXA-48			X	X	X	X	X	X	X	X	X	X	X	X
4X CTX-M-15				X	X	X	X	X	X	X	X	X	X	X
blaCTX-M-15			X											
blaTEM-1A		X												
blaOXA-1			X											
blaKPC-3	X	X												
blaOXA-9		X												
dfrA12				X	X	X	X	X	X	X	X	X	X	X
dfrA5			X											
mph(A)			X	X	X	X	X	X	X	X	X	X	X	X
mph(E)	X		X	X	X	X	X	X	X	X	X	X	X	X
msr(E)	X		X	X	X	X	X	X	X	X	X	X	X	X
qnrS1			X											
2X sul1			X											

sul1	X	X		X	X	X	X	X	X	X	X	X	X	X
sul2			X											
catB3			X											
tet(A)				X	X	X	X	X	X	X	X	X	X	X
blaSHV-182		X												
blaSHV-67			X	X	X	X	X	X	X	X	X	X	X	X
OqxA	X	X	X	X	X	X	X	X	X	X	X	X	X	X
OqxB		X	X	X	X	X	X	X	X	X	X	X	X	X
fosA		X	X	X	X	X	X	X	X	X	X	X	X	X
ΔmgrB	X													
ΔPmrB	X													
GyrA-83I		X	X											
GyrA-83Y				X	X	X	X	X	X	X	X	X	X	X
GyrA-87A				X	X	X	X	X	X	X	X	X	X	X
ParC80I		X	X	X	X	X	X	X	X	X	X	X	X	X
ΔOmpK35	X	X	X											
OmpK36GD		X	X	X	X	X	X	X	X	X	X	X	X	X
IncFIB(pNDM-Mar) + IncHI1B(pNDM-MAR)			X											
IncFIB(pNDM-Mar) + IncHI1B(pNDM-MAR) + repB(R1701)				X	X	X	X	X	X	X	X	X	X	X
IncFIB(pQil)		X	X											
IncFIA(HI1)	X													
IncL			X											
IncR	X													
IncX3	X	X												
Col(BS512)				X	X	X	X	X	X	X	X	X	X	X
Col440II	X													
ColRNAI	X	X												
Yersiniabactin	X		X	X	X	X	X	X	X	X	X	X	X	X
Aerobactin	X													
ΔrmpA2	X													

Virulence Score	4	0	1	1	1	1	1	1	1	1	1	1	1	1

Supplementary Material S4
Summary of control measures
(based on internal documents of Policlinico Umberto I Hospital)

On 6 March 2025, the Director of the Department for Emergency–Admission–Anesthesia–Critical Care at Policlinico Umberto I Hospital in Rome reported with an Official Note to the Health Directorate of the Hospital the detection of 4 positive cases of *Klebsiella pneumoniae* NDM, OXA-48-like in the Sub-intensive Care Unit (S-ICU) during the 7 days preceding the notification. No prior cases of the same microorganism ad ever been detected in that ward before.

Following this communication, the Health Director convened an extraordinary meeting of the Hospital Infection Control Committee on 7 March for a joint assessment of the situation and the definition of actions to be taken. In addition, with Offical Note no. 0011421 of 6 March 2025, she ordered the following preliminary actions:

- suspension of non urgent patient movement into and out of S-ICU until further notice;
- strict observance, monitoring, and documentation of adherence by all staff to healthcare-associated infection (HAI) prevention measures:
    - hand hygiene;
    - cleaning and sanitisation of structures/infrastructure;
    - cleaning and sanitisation of medical devices and of surfaces in patient areas and staff work areas;
- separation of the healthcare staff assigned to patients under isolation;
- active surveillance of contacts according to the indications of the infectious-disease consultant.

During the Hospital Infection Control Committe meeting, the positive cases of *K. pneumoniae* NDM+ OXA-48-like identified in the unit were presented, and an epidemiological investigation was conducted, documenting their movements within the hospital wards before and after detection of the microorganisms.
It was therefore decided to proceed with the following action plan:

1. Maintain isolation precautions for the patients who tested positive, recommending the presence of a dedicated healthcare team;
2. Conduct surveillance of all patients admitted in the S-ICU starting from 15/02/2025, the admission date of the first patient who tested positive;
3. The Unit Director and the Ward Coordinator must ensure the strictest observance of HAI prevention measures and hand hygiene;
4. Perform cleaning and sanitisation of structures/infrastructure;
5. Perform cleaning and sanitisation of medical devices and of surfaces in patient areas and staff work areas;
6. Initiate the clinical-epidemiological investigation, coordinated the Hospital Hygiene Unit and supported by a working group of the Infeciton Control Committee, including environmental sampling

Enhanced active surveillance protocol

For the purpose of initiating surveillance activities, all patients who shared the same healthcare team as the confirmed *K.pneumoniae* NDM+ OXA-48-like cases were defined as contacts.

Ward staff were asked to carry out the tracing of all contacts-,that is, all patients who passed through the S-ICU from 15/02/2025, the admission date of the first patient who later tested positive, up to the discharge date of the last positive patient, 06/03/2025-regardless of the reason for hospitalization, specifying the ward to which they were eventually transferred.
In total, 72 patients passed through the S-ICU between 15/02/2025 and 07/03/2025:

- 4 positive cases, placed in isolation in the ICU
- 1 positive case, placed in isolation in a different ward,
- 6 patients placed under contact isolation in the S-ICU, for whom surveillance with a rectal swab every 48 hours for 7 days after the last contact with a case was arranged,
- 62 patients who were discharged, deceased, or transferred to other wards.

On 10/03/2025, the Health Directorate provided an update on the status of patients who had passed through the S-ICU from 15/02/2025 to 07/03/2025: 30 contacts were reported as currently hospitalized in our facility, of whom 6 in the S-ICU and 24 in other wards.
For patients hospitalized in other wards, the infectious disease specialists ordered precautionary isolation and the collection of a rectal swab, to be repeated-if the result was negative-7 days after transfer from the cluster area (the date of last contact with a positive patient).

Surveillance and enhanced control measures were discontinued when no further cases were detected through rectal swab screening, on March 25.
